# An Exploration of the Utility and Impacts of Implementation Science Strategies by Cancer Registries for Healthcare Improvement: A Systematic Review

**DOI:** 10.34172/ijhpm.8297

**Published:** 2024-10-07

**Authors:** Rob G. Stirling, Angela Melder, Emily Eyles, Mark Reich, Paul Dawkins

**Affiliations:** ^1^Department of Respiratory Medicine, Alfred Health, Melbourne, VIC, Australia.; ^2^Central Clinical School, Faculty of Medicine, Nursing and Health Sciences, Monash University, Melbourne, VIC, Australia.; ^3^Health and Social Care Unit, School of Public Health and Preventative Medicine, Monash University, Melbourne, VIC, Australia.; ^4^School of Public Health and Preventative Medicine, Monash University, Melbourne, VIC, Australia.; ^5^Department of Respiratory Medicine, Middlemore Hospital, Auckland, New Zealand.; ^6^Faculty of Medical and Health Sciences, University of Auckland, Auckland, New Zealand.

**Keywords:** Cancer Registry, Implementation Science, Knowledge, Translation, Quality Improvement, Learning Health System

## Abstract

**Background:**

Cancer data registries are central elements of cancer control programs providing critical insights in measures of performance in cancer healthcare delivery. Evidence to practice gaps in cancer care remain substantial. Implementation science (IS) strategies target gaps between generated research evidence and guideline concordance in delivered healthcare. We performed a systematic review of the utilisation and effectiveness of IS strategies reported by cancer registries.

**Methods:**

A research protocol and literature search were performed seeking studies incorporating implementation strategies utilised by cancer registries for quality improvement. Searches were undertaken in MEDLINE, Embase, CENTRAL, and the grey literature for randomised trials and observational studies. The "Knowledge to Action" (K2A) framework was used to explore implementation gaps in care delivery.

**Results:**

Screening identified 1496 studies, 37 studies identified by title and abstract review, and 9 included for full text review. Studies originated from the United Kingdom, the United States, the Netherlands, and Australia reporting on lung, breast, colo-rectal, and cancer clusters. Registry jurisdictions included 7 national, 4 state, and 4 local registries. Knowledge gap analysis consistently identified monitoring and evaluation of data outcomes in accord with registry primary purpose although limited exploration of the utilisation, translation and re-application of this data. Studies lacked description of strategies describing sustainability of generated knowledge, identification of barriers, knowledge adaptation to local contexts, and the selection, adaptation and implementation of interventions for improvement.

**Conclusion:**

Available studies provide limited literature evidence of the effective utilisation of IS strategies reported by cancer registries for healthcare improvement. A substantial opportunity presents to study the engagement of IS in cancer registry data use to close the evidence practice gap and facilitate data driven improvement in cancer healthcare.

## Introduction

 The development of cancer registries has been described as essential for national cancer control programs targeting reduction in cancer incidence and mortality and improvement in quality of life for cancer patients.^1-4^ Early cancer registries described burdens of cancer prevalence, incidence and survival.^5^ Over time, these roles have expanded to include epidemiologic research, risk factor identification, investigation of cancer clusters, monitoring of impacts of healthcare interventions including screening and primary prevention, measurement of disparities in healthcare equity, policy development, resource planning, and as a tool for quality improvement.^6,7^

**Figure 1:**
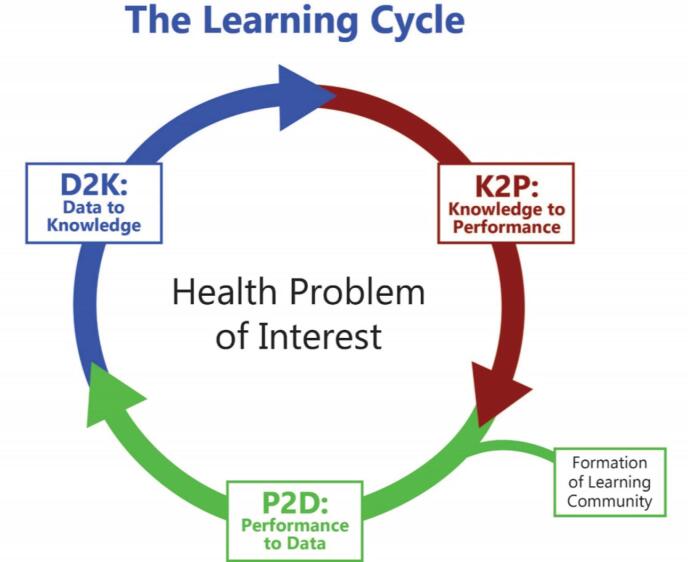
Integration of research evidence (Knowledge to Performance) with clinical performance evidence (Performance to Data) to inform practice improvement (Data to Knowledge) in the Learning Health System.^12^

 The cancer healthcare system has been described as, “a system in crisis where care is not patient-centred, and decisions about care often are not based on the latest scientific evidence.”^8^ Delays of decades have been demonstrated between generation of practice-changing research evidence to effective clinical evidence implementation, described as an “evidence-practice gap.”^9,10^ The learning health system (LHS) strategy was developed to overcome this gap by addressing two key issues.^11^ First, the integration of local, rapidly generated, clinical performance knowledge, with comprehensive research knowledge, gained from systematic literature review (Figure 1).^12^ Second, making this combined evidence available to efficiently and effectively inform practice improvement.^10,13,14^ This knowledge may then inform and drive iterative innovation and practice change to enhance system knowledge and performance.

 Cancer registries and clinical quality registries (CQRs) provide the necessary framework to enable the integration of local, population-specific performance data in cancer management with the external research evidence that informs and updates registry purpose and design. Closing the LHS loop however demands data integration that facilitates the use of curated data to be represented, disseminated, and applied in innovation and implementation for healthcare improvement.^10^

 Implementation science (IS), encompassing dissemination and implementation approaches, provides strategies developed to improve the translation of research knowledge into practice to reduce evidence-practice gaps.^15-18^ Little is known however of the extent of use and practical impacts of IS strategies in the translation of cancer registry data to prompt healthcare improvements or measure improved health-care outcomes.

 We aimed to answer three questions: (1) What evidence is available to describe the use of IS strategies by cancer registries to improve cancer outcomes? (2) What evidence is available of the effectiveness of such IS strategies in utilising cancer registry data? (3) What are the potential opportunities for IS strategies for cancer registries to drive improvement in healthcare outcomes in cancer?

## Methods

 We performed a systematic review, undertaken to explore the characteristics and extent that IS strategies were used in reported research, and to explore the mapping, reporting or discussion of these characteristics and concepts in relation to cancer registry activities.^19^

###  Protocol Registration

 A study protocol was created and registered in the PROSPERO registry of systematic reviews (CRD42021251860).

###  Knowledge Translation Definition

**Figure 2:**
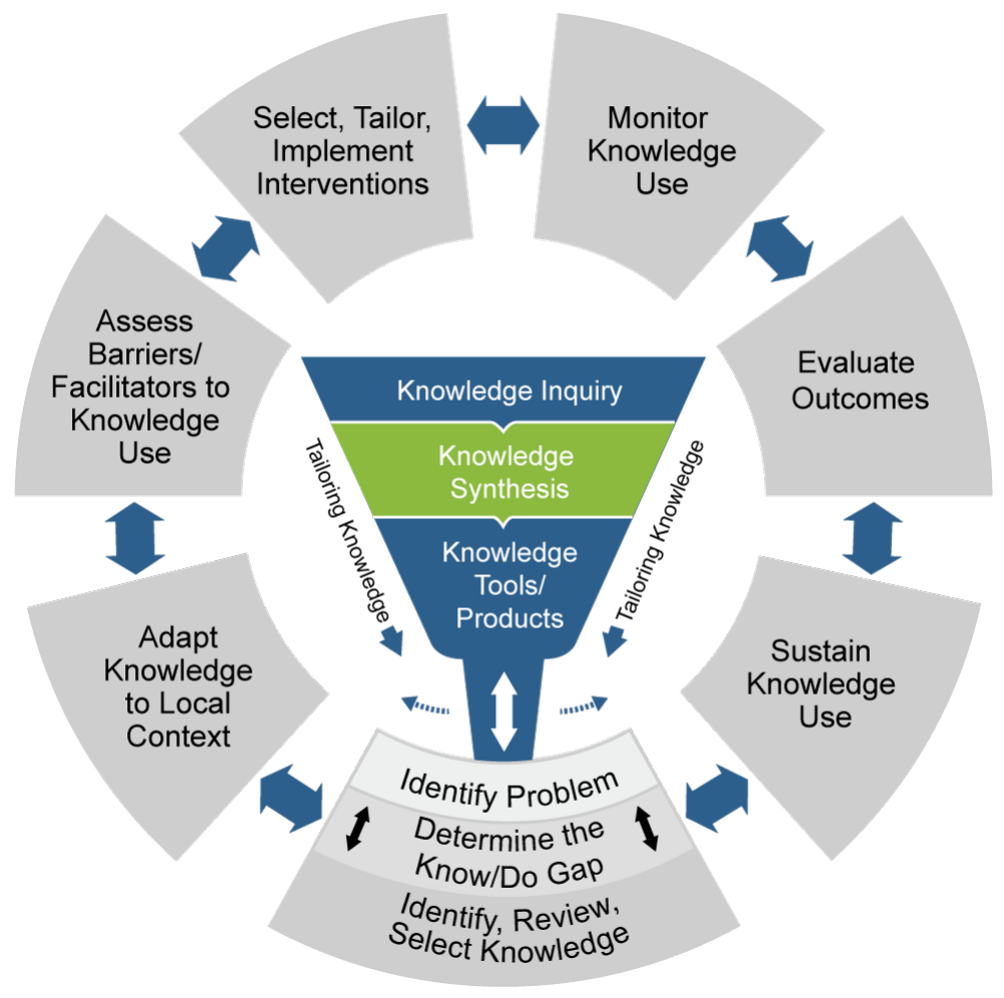
Knowledge Translation: 7 Step Knowledge to Action Framework.^20,21^

 Our definition of knowledge translation was based on the Canadian model^20^: “a dynamic and iterative process that includes the synthesis, dissemination, exchange and ethically sound application of knowledge to improve health, provide more effective health services and products and strengthen the healthcare system,” engaging the 7-step knowledge to action (K2A) framework^21^ (Figure 2).

###  Eligibility Criteria 

####  Study Designs

 Retrospective and prospective studies including, randomized-controlled trials, clinical trials, case control, cohort, observational, follow-up, cross-sectional, qualitative research, systematic reviews, and study protocols were included. Commentaries, editorials, letters, and news articles were excluded from this review process. A search strategy generated in Medline is included in Table S1 (Supplementary file 1).

####  Types of Participants

 The participants included within the scope of this review included any possible knowledge users within cancer research, cancer policy, clinical, quality improvement or consumer communities that may be targets of IS strategies.

####  Included and Excluded Interventions

 Studies including interventions that targeted improvement in cancer patients’ care and outcomes were considered for inclusion. Interventions had to describe an IS intervention and report outcomes using data from a cancer registry. Studies were excluded if they did not identify an IS intervention or outcome.

###  Information Sources

 The searches covered these electronic databases: MEDLINE, Embase, Cochrane, CINAHL, PsycInfo, Web of Science, Scopus, and ProQuest. The searches of the electronic databases were conducted on April 11, 2021.

###  Selection of Sources of Evidence

 Title and abstract screening of imported studies were completed by two reviewers. Discrepancies were reviewed and lack of consensus resolved by a third reviewer. Full-text analysis followed to assess approved abstracts and abstracts that required further information to be considered for inclusion.

###  Evidence of Utilisation of Implementation Science Strategies

 The K2A framework was used as a knowledge translation platform to identify the extent of utilisation of IS strategies and to define evidence gaps.^21,22^ Evidence addressing any of the 7 steps of the K2A framework was sought to inform the evidence gap map (Figure 2). Two reviewers categorised each study according to included K2A steps. Further, we mapped discrete identified IS interventions using the consensus categorisation provided by the Expert Recommendations for Implementing Change (ERIC) project^23^ and further mapped independent strategies to implementation concept clusters using the method of Waltz et al.^24^ These consensus statements enable the definition of complex, diverse, multi-level implementation strategies, effectively improving the specifications and consensus reports of implementation strategies, assisting in the characterisation of discrete strategies published in implementation research.

## Results

**Figure 3:**
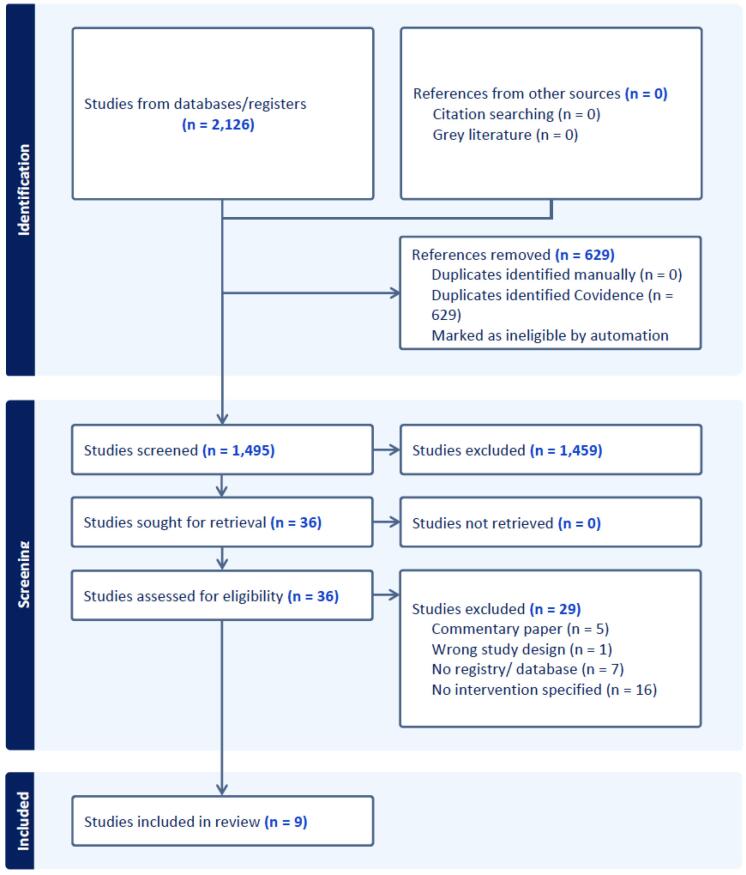
PRISMA Diagram Study Selection. Abbreviation: PRISMA, Preferred Reporting Items for Systematic Reviews and Meta-Analyses.

 The search identified 2126 references, with 1495 remained after duplicate exclusion (Figure 3). Inclusion and exclusion criteria resulted in 9 studies available for study inclusion after full text review,^25-33^ with details of selected studies described in Table 1. Reports emanated from the United Kingdom, the United States, the Netherlands, and Australia, with 4 studies describing national registries and 5 regional or state-based registries. Four studies described efforts in multiple cancers, 4 lung cancer, and 1 breast cancer.

**Table 1:** Study Characteristics

**Study**	**Country**	**Cancer **	**Registry **	**Study Design**	**Participants**	**Intervention Studied**	**Outcome**	**Impacts**
Aveling 2012	UK	Lung	NLCA LUCADA	Ethnographic mixed methods qualitative study	30 paired hospital multidisciplinary lung cancer teams	RP2PR	Nonparticipant observation, interviews and documentary analysis.	5 Factors were identified as important in optimising RP2PR: peer and pairing methods; minimizing logistic burden; structure of visits; independent facilitation; and process credibility.
Beckett 2012	UK	Lung	NLCA	Observational study of NLCA establishment and progress 2005-2009	All audit captured lung cancer registration (140 000)	Establishment and conduct of annual audit	Lung cancer management process and outcome measures.	Histological confirmation rate (64%–76%), the proportion of patients discussed in MDM (78%–94%), proportion of patients having active anti-cancer treatment (43%–59%), surgical resection (9%–14%), and SCLC chemotherapy (58%–66%).
Klaiman 2014	USA	Cancer	Louisiana, New York State and Texas Cancer Registries	Qualitative inventory of cancer registries using literature review, web search and expert opinion to identify best practices of effective registries (positive deviance)	State cancer registry inclusions	Examination, literature review and expert panel discussion to identify best practices of effective’ registries	Evaluation of registries to define the factors that make them effective.	Effective registries were successful in >1 of 6 key areas: data standardisation, transparency, accuracy/completeness of data, participation by providers, financial sustainability, and/or feedback to providers.
Russell 2014	UK	Lung	NLCA	Prospective RCT	30 paired hospital multidisciplinary lung cancer teams	RP2PR	Proportion of patients discussed in a MDM, histological confirmation rate, active treatment rate, surgical resection rate, the proportion of patients with SCLC receiving chemotherapy and the proportion of patients seen by a lung cancer nurse specialist.	Proportion receiving active anti-cancer treatment in the intervention group increased by 5.2% compared with 1.2% in the controls (mean difference 4.1%, 95% CI 0.1–8.2%, *P* = .055). The remainder of NLCA indicators improved consistently in intervention and control groups.
McAlearney 2016	USA	Breast	AMCR	Qualitative research interview and expert panel discussion of barriers and facilitators to implementation of intervention	Hospital- and community-based oncologists and hospital cancer leaders recruited for participation based on medical center affiliation	Intervention designed to increase registration of cancer treatment information	Challenges to implementation included lack of understanding of research evidence, provider time constraints, competing priorities within healthcare organizations, unsupportive information technology, misaligned incentives, organizational and cultural factors.	Tumour treatment detail registration increased from 2.6% to 64%.
Smittenaar 2019	England	Breast	NCRAS RTDS SACT HES	Population level observational data/review	Breast cancer patients receiving systemic anti-cancer therapy	Provision of anti-cancer treatment outcome data, data helpline access and improvement workbook	Early mortality for breast cancer patients treated with curative intent was 0.3%. Impacts of workbooks unreported.	Real world evidence of 30-day mortality confirmed as similar to trial evidence.
Tucker 2019	USA	Cancer, Colorectal Cancer	SEER Medicare KCR	Review	CRC eligible subjects >50 years in Kentucky.	Lay health navigators, academic detailing primary care physicians, assistance in screening scheduling. Mandated CRC insurance screening coverage for age eligible individuals	Proportion of age-eligible adults in Kentucky undergoing either lower colonic endoscopy.	Screening uptake rose from 34.7% in 1999 to 63.7% in 2008.
Van der Hout 2020	The Netherlands	Head and neck, colorectal, breast, Hodgkin or non-Hodgkin lymphoma	NCR PROFILES	Non-blinded, RCT	Cancer survivors in 14 hospitals in the Netherlands (n = 625)	Oncokompas; web-based eHealth application supporting self-management by monitoring general cancer and cancer-specific symptoms and HRQOL, providing personalised feedback to reduce symptom burden and improve HRQOL	Primary outcome was patient activation (knowledge, skills and confidence for self-management).	Patient activation did not differ between intervention control groups over time (6-months follow-up 1.7 (95% CI −0.8 to 4.1; *P* = .41). HRQOL score was significantly improved at 6 months *P* = .048.
Largey 2020	Australia	Lung	VLCR CQR	Prospective quality improvement cohort study	Consecutive patients from 5 participating hospitals (n = 205)	Community of practice forums to identify service gaps, variation drivers and barriers to improvement	Quality improvement process and outcome measures from the VLCR.	There was an increase in proportion of new referrals seen by a specialist within 14 days (74.3% to 84.2%), reduction in variation in timeliness between sites. The proportion of subjects with documented presentation to an MDM (61% to 67%, *P* > .05). No observed effects on timeliness from first specialist appointment to first staging test or PET scan. Trend to increase in supportive care screening documentation (22% to 26.3% *P* = .06).

Abbreviations: NLCA, National Lung Cancer Audit; SEER, Surveillance, Epidemiology, and End Results; LUCADA, National Lung Cancer Audit; AMCR, Academic Medical Centre Registry; KCR, Kentucky Cancer Registry; NCRAS, National Cancer Registration and Analysis Service; SACT, Systemic Anti-Cancer Therapy; HES, Hospital Episode Statistics; MDM, multidisciplinary meeting; SCLC, small cell lung cancer; NCR, Netherlands Cancer Registry; PROFILES, Patient Reported Outcomes Following Initial Treatment and Long term Evaluation of Survivorship; VLCR, Victorian Lung Cancer Registry; CQR, clinical quality registry; CRC, colorectal cancer; RTDS, National Radiotherapy Dataset; RP2PR, reciprocal peer-to-peer review; RCT, randomised controlled trial; HRQOL, health related quality of life; PET, positron emission tomography.

###  Summary of Implementation Science interventions

 Becket et al reported outcomes of the UK National Lung Cancer Audit (NLCA) which disseminates registry outcomes through targeted reports to key institutional stakeholders, and through presentation at local, national and international meetings undertaken by the project team.^26^ The authors identified the need to establish stakeholder trust within the report content by confirming data completeness and risk adjustment for key measures including deprivation, comorbidity and casemix. The report linked stakeholders to a quality improvement toolkit, providing a targeted checklist of eight key areas from the report for use by multidisciplinary teams.^34^

 The Improving Lung Cancer Outcomes (ILCOP) study group reported on a randomised controlled trial (RCT) in which reciprocal peer-to-peer review (RP2PR) was conducted amongst 30 paired multidisciplinary teams using UK NLCA registry data.^28^ Quality improvement facilitators provided a structured quality improvement planning template and education around models for improvement resulting in 67 quality improvement plans being implemented, resulting in a modest increase in active treatment in intervention groups (n = 31) of 5.2% compared with 1.2% in control groups (*P* = .055). The remainder of study measures improved similarly in intervention and control cohorts. Mean patient experience scores were not significantly impacted although improvement was observed for 5 of the teams with the worst baseline scores (*P* =.001).

 Aveling et al reported an ethnographic study of the RP2PR process from the ILCOP study, describing the improvement programme attempting to identify the implementation elements that appeared to optimise the function of this model.^25^ Observation, interviews and documentary analysis was undertaken, and 5 identified core process elements were important in enhancing this model: peers and pairing methods, minimising logistic burden, structure of visits, independent facilitation, and credibility of the process. RP2PR impacts were maximised when organised, undertaken in a safe learning environment, where credibility, implementation and impacts were promoted.

 Klaiman et al evaluated registries to identify the tools and strategies associated with positive deviation in quality improvement, value-based purchasing and stakeholder reporting on quality of care.^27^ The project group conducted web search, literature review and direct interviews with experts from the Louisiana, New York State and Texas Cancer Registries. Structural and functional diversity between registries made the identification of registry characteristics likely to deliver positive impacts difficult to identify. Six key themes of registry function however were identified in effective registries including data standardisation, transparency, accuracy and completeness of data, provider participation, financial sustainability, and feedback to providers.

 McAlearney et al reported on tumour registry capture of breast cancer adjuvant therapies.^29^ The authors identified barriers including lack of understanding of current research by clinicians and hospital managers, clinician time limitations, concurrent priorities within healthcare organisations, unsupportive information technology, incentive misalignment, and organisational/cultural factors. Four internal threats to implementation were identified including: loss of the innovation champion; a lack of shared commitment to implementation between different stakeholder groups: inconsistent management support of the implementation; and resource insecurity related to the concurrent implementation of an electronic medical record.

 Smittenaar et al described the use of National Cancer Registration and Analysis Service (NCRAS) at Public Health England to provide real world validation of RCT results, adverse event reporting and to describe treatment adherence and variation in the context of breast cancer.^30^ Participating centres were encouraged to engage quality improvement in exploration of variation in early (30-day) mortality and to identify improvement opportunities, by providing workbooks supporting mortality and morbidity meetings and providing early warnings regarding differing toxicities between RCTs and real world care.

 Tucker et al addressed the problem of high colorectal cancer prevalence and low screening rates within the Kentucky Cancer Registry.^31^ Public health advocacy was initiated resulting in mandated health insurance company coverage to ensure screening colonoscopy was remunerated for age-eligible individuals. Health navigators provided targeted education to the public, identified and managed cultural barriers to screening and provided logistic support to facilitate appointment scheduling, while focused education programs targeted primary care physicians to promote widespread screening uptake. Screening rates following this intervention rose from 34.7% in 1999 to 63.7% in 2008.

 Van der Hout et al reporting on the Dutch PROFILES registry, aimed to provide a fully web-based behavioural intervention technology (Oncokompas) for use independently by cancer survivors. The tool incorporated measure, learn and act components supporting knowledge, skills and confidence in self-management aimed at improving symptoms and health related quality of life (HRQOL) by responding to symptom burden with a series of supportive care options.^32^ The study included a randomised controlled design including multiple cancer types with the primary outcome of Patient Activation Measure. Trial enrolment included 21% of available cancer survivors, with 52% using Oncokompas as proposed. Although the primary outcome of patient activation was not met, most tumour groups had significant and meaningful improvements in HRQOL and tumour specific symptoms.

 Largey et al reported a quality improvement collaborative,^33^ engaging patient advocates, clinicians, hospital administration and governance, redesign experts and researchers to drive site specific innovation development and solution sharing, targeting national Optimal Care Pathway objectives for lung cancer^35^ across 5 hospitals, sourcing Victorian Lung Cancer Registry data.^36^ Marked improvements in timeliness of referral to first specialist appointment (median [interquartile range]from 6 [0-15] to 4 [1-10] days), proportion seen in specialist care within 14 days (74.3% to 84.2%) and proportion reviewed in a multidisciplinary meeting (61% to 67%) were observed.

###  Mapping Utilised Implementation Science Strategies

 Based on the K2A framework, we identified that studies routinely engaged monitoring and evaluation of data outcomes consistent with primary function of the registry and provided guidance in problem identification consistent with the project objectives. There was however minimal discussion of approaches to assessment of barriers to knowledge use, selecting, tailoring and implementation of interventions to address barriers and scant description of strategies to sustain knowledge translation behavioural changes (Table 2).

**Table 2:** Evidence of Utilisation of the Knowledge to Action Framework Steps

**Study**	**Setting**	**Cancer Type**	**K2A Framework Steps**						
			**Monitor Knowledge Use**	**Evaluate Outcomes of Knowledge Use**	**Developing Mechanisms to Sustain Knowledge Use**	**Identifying the Problem, and Identifying, Reviewing and Selecting Knowledge**	**Adapting Knowledge to Local Context**	**Assessing Barriers & Facilitators to Knowledge Use**	**Selecting, Tailoring and Implementing Intervention to Address Barriers to Knowledge Use**
Aveling 2012	National	Lung	-	-	-	+	+	+	+
Beckett 2012	National	Lung	+	+	+	+	-	-	-
Klaiman 2014	Regional/state	All cancer	+	-	+	-	-	-	-
Russell 2014	National	Lung	+	+	-	+	+	+	+
McAlearney 2016	Regional	Breast	+	+	-	+	+	+	+
Smittenaar 2019	National	All cancer	+	+	-	+	+	-	-
Tucker 2019	Regional/state	All cancer/CRC	+	+	+	+	+	+	+
Van der Hout 2020	Regional	Head and neck, colorectal, breast, lymphoma	+	+	-	-	-	-	-
Largey 2020	Regional	Lung	+	+	-	+	+	+	+
Total			8	7	3	7	6	5	5

Abbreviations: CRC, colorectal cancer; K2A, knowledge to action.

 We identified utilisation of 43 of 73 implementation strategies defined and summarised from the ERIC study^23^ (Table 3). Broadly utilised strategies included audit and feedback, development and delivery of education materials, project facilitation and convening expert advisory groups. Infrequently used strategies included engagement of governance, opinion of patients and families, ongoing consultation, enhancement of quality monitoring systems, relay of clinical data to providers, creation of financial incentives to enhance participation and the use of mandate for change.

**Table 3:** Intervention Strategies Utilised and ERIC Category Correlates

**Study**	**Reported Study Implementation Strategies**	**ERIC Discrete Implementation Strategies**^22^	**Implementation Concept Cluster**^23^
Aveling 2012	Nonparticipant observation. Semi structured interviews. Documentary analysis.	Purposefully re-examine the implementation. Conduct local need assessment. Conduct cyclical small tests of change.	A
Beckett 2012	Multidisciplinary workshops. Collaboration with clinical effectiveness unit. Expert reference group including patient/carer representation. Create clinical dataset. Online data entry portal. Provide telephone helpdesk. Centralised data repository. Central data analysis. Provide casemix adjusted data reports to clinicians.	Audit and provide feedback. Develop and implement tools for quality monitoring. Develop and organize quality monitoring systems. Develop a formal implementation blueprint. Stage implementation scale up. Obtain and use patients/consumers and family feedback. Facilitation. Provide local technical assistance. Use data experts. Use data warehousing techniques. Build a coalition. Use advisory boards and workgroups. Promote network weaving. Work with educational institutions. Facilitate relay of clinical data to providers. Involve patients/consumers and family members.	ACDEFG
Klaiman 2014	Develop expert panel research team to define validated, objective criteria for identifying effective registries in key clinical areas and the factors that make them effective.	Use of advisory boards and workgroups	D
Russell 2014	Introductory educational workshop. Facilitated peer to peer visits. Observation of MDM. Discussion of MDM function. Audit data review. Patient experience questionnaire. Focus of improvement workshop. Facilitated QI template. Follow up email, telephone and visit. Web based collaborative teleconferences. Face to face redesign review workshops.	Audit and provide feedback. Purposefully re-examine the implementation. Develop and implement tools for quality monitoring. Develop and organize quality monitoring systems. Conduct local need assessment. Facilitation. Provide local technical assistance. Organize clinician implementation team meetings. Conduct local consensus discussions. Capture and share local knowledge. Use advisory boards and workgroups. Use an implementation advisor. Visit other sites. Provide ongoing consultation. Make training dynamic. Conduct educational meetings. Conduct educational outreach visits. Create a learning collaborative. Facilitate relay of clinical data to providers.	ABDEF
McAlearney 2016	In person interviews with key informants. Semi structured interview guides. Coding dictionary. Dynamic coding evaluation. Convene expert panel. Soliciting feedback. Regular investigator consensus discussions.	Assess for readiness and identify barriers and facilitators. Purposefully re-examine the implementation. Conduct local need assessment. Conduct cyclical small tests of change. Tailor strategies. Conduct local consensus discussion.	ACD
Smittenaar 2019	Study risk factors early mortality after SACT. Provide early mortality workbook to clinicians. Provide SACT helpdesk.	Audit and provide feedback. Develop and implement tools for quality monitoring. Capture and share local knowledge. Develop educational materials. Facilitate relay of clinical data to providers.	ADEF
Tucker 2019	Advocate for insurance coverage for age eligible colonoscopy for CRC cancer screening. Use lay health navigators to overcome cultural barriers. Persuade primary care providers to recommend screening. Schedule CRC screening appointments. Measuring changes in the CRC incidence rate over time.	Conduct local need assessment. Audit and provide feedback. Develop and organize quality monitoring systems. Facilitation. Tailor strategies. Organize clinician implementation team meetings. Conduct educational outreach visits. Intervene with patients/consumers to enhance uptake and adherence. Alter incentive/allowance structures.	ABCEGH
Van der Hout 2020	Develop web-based eHealth survivor self-management application. Provide feedback and patient-specific advice on self-management. Data measures linked to tailored feedback. Healthcare provider invites participants. Central data storage. Longitudinal reassessment.	Audit and provide feed. Develop and implement tools for quality monitoring. Develop and organize quality monitoring systems. Centralize technical assistance. Use data experts. Use data warehousing techniques. Develop educational materials. Distribute educational materials. Remind clinicians. Involve patients/consumers and family members. Intervene with patients/consumers to enhance uptake and adherence.	ABCEFG
Largey 2021	Convene multidisciplinary evaluation and solution committee. Stakeholder workshops. Baseline process evaluation. Variation and barrier analysis. QI toolbox engagement. Service redesign modelling. Root cause analysis. Targets prioritised for improvement. Design solutions generated. Community of practice forums. Collaborative learning. QI education and support. Shared problem identification and solution sharing. Web based data capture. Secure central data management. Defined performance indicators.	Build a coalition. Conduct educational meetings. Conduct local consensus discussions. Create a learning collaborative. Facilitation. Conduct ongoing training. Conduct educational meetings. Develop educational materials. Capture and share local knowledge. Conduct local consensus discussions. Create a learning collaborative. Build a coalition. Develop educational materials. Distribute educational materials. Identify and prepare champions. Involve executive boards. Involve patients/consumers and family members. Obtain and use patients/consumers and family feedback. Audit and provide feedback. Promote adaptability. Promote network weaving. Use data experts. Use data warehousing techniques.	ABCDEG

Abbreviations: SACT, Systemic Anti-Cancer Therapy; CRC, colorectal cancer; ERIC, Expert Recommendations for Implementing Change; QI, Quality Improvement; MDM, multidisciplinary meeting.

 Reported studies frequently utilised implementation strategies from categories including use evaluative and iterative strategies, training and education of stakeholders, adapting and tailoring to context and developing stakeholder interrelationships (Supplementary file 1, Table S2). Infrequently utilised implementation categories included utilisation of financial strategies and change to infrastructure.

## Discussion

###  Utilisation of Implementation Science Strategies

 We found only limited evidence of systematic utilisation of IS strategies by cancer registries in order to improve healthcare decision-making for quality improvement. Studies included national and state-based registries describing lung, breast, colorectal and multiple cancers. When we mapped IS strategies from included studies to the K2A framework it revealed that monitoring and evaluation of data outcomes was common, however there was minimal description of strategies for sustaining behavioural interventions, adaptation of knowledge interventions, assessment of barriers to implementation, and selection of effective interventions for implementation.

 The most commonly used implementation concept clusters included the use of evaluative and iterative strategies for data evaluation in 8 of 9 reports (8/9), development of stakeholder interrelationships (6/9), training and education of stakeholders (6/9) and adapt and tailoring to context (5/9). Infrastructural change (0/9) and the utilisation of financial strategies (1/9) were rarely used concept clusters.

 In a broad review of registry capability including cancer, orthopaedic, obstetric and cardiovascular registries, Klaiman et al drew the disconcerting conclusion that state cancer registries, “exhibited the fewest innovations to enhance quality improvement applications,”^27^ potentially inviting cancer registries to more actively engage reported data to monitor knowledge use; evaluate outcomes of knowledge use and to identify problems and opportunities to review and select knowledge for quality improvement.

###  Effectiveness of Implementation Science Strategies

 Two studies reported clinical communities as quality improvement collaboratives.^28,33^ Van der Hout reported overall improvement in HRQOL, with no impact on the primary outcome of patient activation, while there was some minor improvement in the overall measures.^29^ Largey reported non-significant increases in receipt of active treatment +5.2% (*P* = .055) and multidisciplinary meeting presentation, +6% (*P* = .065) but no difference in the overall panel of measures.^33^ No data was available to confirm sustained improvement beyond the trial periods.

###  The Learning Health System

 Abernethy et al describe a rapid learning healthcare model using clinically developed healthcare data in which the healthcare system adapts by: (1) routinely and iteratively collecting data in a planned and strategic manner; (2) analysing captured data; (3) generating evidence through observational analysis of existing and prospective study data; (4) implementing new insights into subsequent clinical care; (5) evaluating outcomes of changes in clinical practice; and (6) generating new hypotheses for investigation.^37^ Key to the effectiveness of such a system is linkage and integration of disparate clinical cancer healthcare performance evidence held within repositories including electronic medical records, CORs, state and national cancer surveillance registries, insurance and funding bodies, civic and administrative datasets. Systematic review of effectiveness of disease registries in LHSs suggests broad patient benefits including better symptom detection, shorter cancer treatment waiting times, and better evidence-based care delivery.^11^ Benefits to clinician-patient encounters include enhanced symptom reporting, health status and HRQOL,^38^ while benefits to health system performance have included identification and management of process barriers and resistance to change, and alignment of system priorities for enhanced best practice care delivery.^39^

###  Sustainability of Change

 Sustainability in healthcare improvement practice relates to the ability to ensure persisting behavioural change within a system. Inducement, incentivisation and data transparency provide motivation to sustainability through bonus programs, preferred provider network status, and reimbursement.^31^ Transparent public reporting of provider and hospital level data have been highly effective in driving change in cardiac registry outcomes,^39^ but as yet has had limited translation to cancer registry activity. Legislatively mandated cancer registry participation is exemplified in Denmark and the United Kingdom, both providing substantial outcome improvement.^40,41^ To date, little evidence exists on sustainability of knowledge translation in the absence of clear incentivisation and inducement.^42^

###  Cross-sector Partnerships

 Lawler et al reported on The Northern Ireland Cancer Registry and described the important ability to describe outcomes across the complete patient journey, achieved by linkage of social and health service delivery data^43^ and the capture of patient reported outcome measures. This initiative has seen the development of strong cross-sectoral partnerships uniting patients, investigators, healthcare professionals, hospital networks, bio-industry, and government initiatives. Key linkage partners include the Northern Ireland Biobank, the pharmaceutical industry through the Northern Ireland Cancer Trials Centre, and consumer forums providing strong patient support shaping personal and public engagement in research.

###  Data Linkage

 Cancer registries continue to evolve in both function and scope. Functional efficiencies are being gained through the use of more effective data capture using probabilistic record linkage to describe patient pathways better, especially when treatments may be delivered in multiple institutions over protracted periods of time.^31^ Natural language processing using open-source information extraction algorithms within electronic medical records have the capacity to increase data linkage, extraction efficiency, case ascertainment and timeliness.^44,45^ Registry scope is increasing with users projecting findings from covered populations to similar neighbouring populations, and projecting predicted findings to future populations. Registries may also link tumour biobank and pathology datasets enabling access to comprehensive molecular profiling, confirmation of real-world effectiveness of clinical trial data and delivery of precision cancer medicine.^46-48^

###  Consumer Participation

 The engagement of patients as key players in translational research has been embraced by community organisations including the Association of Cancer Online Resources and Patients Like Me (https://www.patientslikeme.com/). By promoting participatory medicine and the dissemination and exchange of information, social and patient networks have the capacity to facilitate patient access to relevant information, promulgate clinical trial outcomes, and facilitate clinical trial recruitment, and use online and personalised feedback to enhance clinical decision-making and impact outcomes. The feasibility of incorporation of patient reported outcome measures in cancer registries has been demonstrated,^49^ although the full attributable benefits remain to be demonstrated.

###  Research Potential

 The enhancement of cancer registries as research infrastructures to drive clinical decision-making, quality, value and cost effectiveness of care is likely to be further enhanced by increased capture and linkage of informative data sources. These data may include environmental exposure, infection, lifestyle, diet activity, health behaviours, genomic and molecular Biobanks.^46^ Cancer registries are key multifunctional data repositories with roles in cancer quality improvement in supporting performance knowledge utilisation and as a tool for IS engagement.

###  Cost Effectiveness

 Cost, cost effectiveness, cost constraint and value are key outcome measures in cancer care. The ability to accurately evaluate cost and comparative effectiveness of available treatments is achievable using registry function and of the utmost importance.^50,51^ An analysis of clinical disease quality registries suggested they are a cost-effective means of quality improvement, providing estimated overall return on investment of 1.6-5.5 multiples of the initial investment costing.^52^

###  Limitations 

 This systematic review contains a wide range of study designs with limited capacity for description of comparability of study quality and a risk of bias assessment. The lexicon of knowledge translation is rapidly evolving and there is potential selection bias by failing to identify all potential citations relevant to the search. Second, there is a risk of selection and publication bias in the failure of publication of negative studies. Third, the structure and capability of registry datasets to describe clinical performance and drive implementation may be determined by data content and characteristics included within registries and these characteristics are not well described in publications describing registry use. Further, clinical, research and academic teams may focus on knowledge generation rather than the full context of healthcare implementation strategies which may be undertaken by health system administration teams and therefore may not be captured in academic publications. It is further possible that other non-clinical healthcare policies have been enacted and implemented on the basis of this knowledge, yet not described in these publications, such as, health literacy training and education, legislation, remuneration, insurance, and organisational alignment in these jurisdictions. IS is an emerging field with significant international variation in definition and terminology; we attempted to overcome this by developing the search strategy with a multidisciplinary team of researchers including a librarian with search strategy development expertise.

## Conclusion

 We found limited evidence of utilisation of IS strategies to improve decision-making in the context of cancer registries described as “essential to the support of national cancer control programs,” designed with the intention to reduce cancer incidence and mortality and improve the quality of life of cancer patients. Cancer registries may however provide the critical necessary infrastructural support to drive quality improvement and establish the basis for cancer LHSs. The application of effective IS strategies in cancer registry function has the potential to improve cancer healthcare decision-making and cancer outcomes.

## Ethical issues

 Not applicable.

## Conflicts of interest

 Authors declare that they have no conflicts of interest.

## Supplementary files

Supplementary file 1 contains Tables S1-S2.

## References

[1] World Health Organization (WHO). National Cancer Control Programmes: Policies and Managerial Guidelines. Geneva: WHO; 2002.

[2] Parkin DM (2008). The role of cancer registries in cancer control. Int J Clin Oncol.

[3] Armstrong BK (1992). The role of the cancer registry in cancer control. Cancer Causes Control.

[4] Forsea AM (2016). Cancer registries in Europe-going forward is the only option. Ecancermedicalscience.

[5] White MC, Babcock F, Hayes NS (2017). The history and use of cancer registry data by public health cancer control programs in the United States. Cancer.

[6] Roder DM, Fong KM, Brown MP, Zalcberg J, Wainwright CE (2014). Realising opportunities for evidence-based cancer service delivery and research: linking cancer registry and administrative data in Australia. Eur J Cancer Care (Engl).

[7] Wei W, Zeng H, Zheng R (2020). Cancer registration in China and its role in cancer prevention and control. Lancet Oncol.

[8] Committee on Improving the Quality of Cancer Care: Addressing the Challenges of an Aging P, Board on Health Care S, Institute of M. In: Levit L, Balogh E, Nass S, Ganz PA, eds. Delivering High-Quality Cancer Care: Charting a New Course for a System in Crisis. Washington (DC): National Academies Press (US); 2013. 24872984

[9] Morris ZS, Wooding S, Grant J (2011). The answer is 17 years, what is the question: understanding time lags in translational research. J R Soc Med.

[10] Guise JM, Savitz LA, Friedman CP (2018). Mind the gap: putting evidence into practice in the era of learning health systems. J Gen Intern Med.

[11] Enticott J, Johnson A, Teede H (2021). Learning health systems using data to drive healthcare improvement and impact: a systematic review. BMC Health Serv Res.

[12] Friedman CP, Rubin JC, Sullivan KJ (2017). Toward an Information Infrastructure for Global Health Improvement. Yearb Med Inform.

[13] Friedman CP, Wong AK, Blumenthal D (2010). Achieving a nationwide learning health system. Sci Transl Med.

[14] Enticott JC, Melder A, Johnson A (2021). A learning health system framework to operationalize health data to improve quality care: an Australian perspective. Front Med (Lausanne).

[15] Woolf SH, Purnell JQ, Simon SM (2015). Translating evidence into population health improvement: strategies and barriers. Annu Rev Public Health.

[16] Lobb R, Colditz GA (2013). Implementation science and its application to population health. Annu Rev Public Health.

[17] Rositch AF, Unger-Saldaña K, DeBoer RJ, Ng’ang’a A, Weiner BJ (2020). The role of dissemination and implementation science in global breast cancer control programs: frameworks, methods, and examples. Cancer.

[18] Melder A, Robinson T, McLoughlin I, Iedema R, Teede H (2020). An overview of healthcare improvement: unpacking the complexity for clinicians and managers in a learning health system. Intern Med J.

[19] Munn Z, Peters MDJ, Stern C, Tufanaru C, McArthur A, Aromataris E (2018). Systematic review or scoping review? Guidance for authors when choosing between a systematic or scoping review approach. BMC Med Res Methodol.

[20] Canadian Institutes of Health Research (CIHR). Guide to Knowledge Translation Planning at CIHR: Integrated and End-of-Grant Approaches. Ottawa: CIHR; 2015.

[21] Graham ID, Logan J, Harrison MB (2006). Lost in knowledge translation: time for a map?. J Contin Educ Health Prof.

[22] Straus SE, Tetroe JM, Graham ID (2011). Knowledge translation is the use of knowledge in health care decision making. J Clin Epidemiol.

[23] Powell BJ, Waltz TJ, Chinman MJ (2015). A refined compilation of implementation strategies: results from the Expert Recommendations for Implementing Change (ERIC) project. Implement Sci.

[24] Waltz TJ, Powell BJ, Matthieu MM (2015). Use of concept mapping to characterize relationships among implementation strategies and assess their feasibility and importance: results from the Expert Recommendations for Implementing Change (ERIC) study. Implement Sci.

[25] Aveling EL, Martin G, Jiménez García S (2012). Reciprocal peer review for quality improvement: an ethnographic case study of the improving lung cancer outcomes project. BMJ Qual Saf.

[26] Beckett P, Woolhouse I, Stanley R, Peake MD (2012). Exploring variations in lung cancer care across the UK--the ‘story so far’ for the National Lung Cancer Audit. Clin Med (Lond).

[27] Klaiman T, Pracilio V, Kimberly L, Cecil K, Legnini M (2014). Leveraging effective clinical registries to advance medical care quality and transparency. Popul Health Manag.

[28] Russell GK, Jimenez S, Martin L, Stanley R, Peake MD, Woolhouse I (2014). A multicentre randomised controlled trial of reciprocal lung cancer peer review and supported quality improvement: results from the improving lung cancer outcomes project. Br J Cancer.

[29] McAlearney AS, Walker DM, Livaudais-Toman J, Parides M, Bickell NA (2016). Challenges of implementation and implementation research: learning from an intervention study designed to improve tumor registry reporting. SAGE Open Med.

[30] Smittenaar R, Bomb M, Rashbass J, Kipps E, Dodwell D (2019). Early breast cancer in England: evidence into practice: what can national cancer registration and analysis service datasets tell us?. J Cancer Policy.

[31] Tucker TC, Durbin EB, McDowell JK, Huang B (2019). Unlocking the potential of population-based cancer registries. Cancer.

[32] van der Hout A, van Uden-Kraan CF, Holtmaat K (2020). Role of eHealth application Oncokompas in supporting self-management of symptoms and health-related quality of life in cancer survivors: a randomised, controlled trial. Lancet Oncol.

[33] Largey G, Briggs P, Davies H (2021). Victorian lung cancer service redesign project: impacts of a quality improvement collaborative on timeliness and management in lung cancer. Intern Med J.

[34] Quality improvement toolkit for lung cancer services. https://nlcastorage.blob.core.windows.net/misc/AR_2019_QIToolkit.pdf. 2020.

[35] Cancer Council Victoria. Optimal Care Pathway for People with Lung Cancer. Cancer Council Victoria; 2016.

[36] Stirling RG, Evans SM, McLaughlin P (2014). The Victorian Lung Cancer Registry pilot: improving the quality of lung cancer care through the use of a disease quality registry. Lung.

[37] Abernethy AP, Etheredge LM, Ganz PA (2010). Rapid-learning system for cancer care. J Clin Oncol.

[38] Smith SK, Rowe K, Abernethy AP (2014). Use of an electronic patient-reported outcome measurement system to improve distress management in oncology. Palliat Support Care.

[39] Hannan EL, Cozzens K, King SB 3rd, Walford G, Shah NR (2012). The New York State cardiac registries: history, contributions, limitations, and lessons for future efforts to assess and publicly report healthcare outcomes. J Am Coll Cardiol.

[40] Jakobsen E, Green A, Oesterlind K, Rasmussen TR, Iachina M, Palshof T (2013). Nationwide quality improvement in lung cancer care: the role of the Danish Lung Cancer Group and Registry. J Thorac Oncol.

[41] Khakwani A, Rich AL, Powell HA (2013). Lung cancer survival in England: trends in non-small-cell lung cancer survival over the duration of the National Lung Cancer Audit. Br J Cancer.

[42] Tricco AC, Ashoor HM, Cardoso R (2016). Sustainability of knowledge translation interventions in healthcare decision-making: a scoping review. Implement Sci.

[43] Lawler M, Gavin A, Salto-Tellez M (2016). Delivering a research-enabled multistakeholder partnership for enhanced patient care at a population level: the Northern Ireland Comprehensive Cancer Program. Cancer.

[44] Ling AY, Kurian AW, Caswell-Jin JL, Sledge GW, Jr Jr, Shah NH, Tamang SR (2019). Using natural language processing to construct a metastatic breast cancer cohort from linked cancer registry and electronic medical records data. JAMIA Open.

[45] Ford E, Carroll JA, Smith HE, Scott D, Cassell JA (2016). Extracting information from the text of electronic medical records to improve case detection: a systematic review. J Am Med Inform Assoc.

[46] Dillner J (2015). A basis for translational cancer research on aetiology, pathogenesis and prognosis: guideline for standardised and population-based linkages of biobanks to cancer registries. Eur J Cancer.

[47] Malone ER, Oliva M, Sabatini PJB, Stockley TL, Siu LL (2020). Molecular profiling for precision cancer therapies. Genome Med.

[48] Langseth H, Luostarinen T, Bray F, Dillner J (2010). Ensuring quality in studies linking cancer registries and biobanks. Acta Oncol.

[49] Ashley L, Jones H, Thomas J (2013). Integrating patient reported outcomes with clinical cancer registry data: a feasibility study of the electronic patient-reported outcomes from cancer survivors (ePOCS) system. J Med Internet Res.

[50] Winn AN, Ekwueme DU, Guy GP Jr, Neumann PJ (2016). Cost-utility analysis of cancer prevention, treatment, and control: a systematic review. Am J Prev Med.

[51] Greenberg D, Earle C, Fang CH, Eldar-Lissai A, Neumann PJ (2010). When is cancer care cost-effective? A systematic overview of cost-utility analyses in oncology. J Natl Cancer Inst.

[52] Lee P, Chin K, Liew D (2019). Economic evaluation of clinical quality registries: a systematic review. BMJ Open.

