# Supplementary material for: An Exploration of the Utility and Impacts of Implementation Science Strategies by Cancer Registries for Healthcare Improvement: A Systematic Review
Source: Int J Health Policy Manag. 2024 Oct 7;13:8297. doi: 10.34172/ijhpm.8297 (PMC11549572; doi:10.34172/ijhpm.8297)
Supplement: Supplementary file 1 — contains Tables S1-S2. [file ijhpm-13-8297-s001.pdf]

Article title: An Exploration of the Utility and Impacts of Implementation Science Strategies by Cancer Registries for Healthcare Improvement: A Systematic Review

Journal name: International Journal of Health Policy and Management (IJHPM)

Authors' information: Rob G. Stirling[1,2]*, Angela Melder[3], Emily Eyles[4], Mark Reich[4], Paul Dawkins[5,6]

[1]Department of Respiratory Medicine, Alfred Health, Melbourne, VIC, Australia.

[2]Central Clinical School, Faculty of Medicine, Nursing and Health Sciences, Monash University, Melbourne, VIC, Australia.

[3]Health and Social Care Unit, School of Public Health and Preventative Medicine, Monash University, Melbourne, VIC, Australia.

[4]School of Public Health and Preventative Medicine, Monash University, Melbourne, VIC, Australia.

[5]Department of Respiratory Medicine, Middlemore Hospital, Auckland, New Zealand.

[6]Faculty of Medical and Health Sciences, University of Auckland, Auckland, New Zealand.

***Correspondence to:** Rob G. Stirling; Email: r.stirling@alfred.org.au

Citation: Stirling RG, Melder A, Eyles E, Reich M, Dawkins P. An exploration of the utility and impacts of Implementation science strategies by cancer registries for healthcare improvement: a systematic review. Int J Health Policy Manag. 2024;13:8297. doi:10.34172/ijhpm.8297

Supplementary file 1

Table S1. Medline Search Strategy

1.	seer program/ or "national program of cancer registries"/
2.	(registries/ or Databases, Factual/) and exp Neoplasms/
3.	((registries or registry or register or registers) adj2 (cancer* or tumo?r* or neoplas* or carcinoma* or malignan* or adenocarcinoma* or sarcoma* or lymphoma* or leukemi* or blastoma* or oncolog* or adenoma* or chondrosarcoma* or osteosarcoma* or rhabdomyosarcoma* or astrocytoma* or ependymoma* or glioma* or neuroblastoma* or medulloblastoma* or oligodendroglioma* or pheochromocytoma* or retinoblastoma* or cholangiocarcinoma* or melanoma* or mesothelioma* or pheochromocytoma* or paraganglioma* or craniopharyngioma* or esthesioneuroblastoma* or myeloma* or astrocytoma or ependymoma or glioma)).mp.
4.	(regist*4 adj2 (quality or clinical quality or disease quality or what works)).mp. and exp neoplasms/
5.	seer program*.mp.
6.	1 or 2 or 3 or 4 or 5

7.	translational medical research/ or diffusion of innovation/ or information dissemination/ or evidenced-based practice/
8.	((knowledge adj3 (translat* or disseminat* or implement* or exchang* or application* or transfer* or mobili* or diffus* or uptak* or "up tak*")) or ((research or findings) adj3 (translat* or disseminat* or implement* or diffus* or incorporat*))).mp.
9.	(("use" or "us?age" or "using" or utili* or application* or applying or "'taking up or take up or 'taken up" or uptak* or "up tak*" or implement*) adj3 (research result* or research* finding* or clinical research or medical research or health research or scientific research or knowledge or evidence or innovation*)).mp.
10.	(application adj2 (knowledge or research or findings or evidence or science)).mp.
11.	(("into practice" or "in to practice" or 'into action' or "in to action" or "applied in practice" or "into routine practice" or "in to routine practice") adj5 (knowledge or research or findings or evidence or science)).mp.
12.	((Knowledge adj2 (interaction or engagement or applying or adoption)) or (adopt* adj2 (innovation* or evidence or research))).mp.
13.	(Implementation science or dissemination science or implementation research or dissemination research).mp.
14.	("implementation and dissemination" or "dissemination and implementation").mp.
15.	"what we know and what we do".mp.
16.	((implement* adj2 (disseminat* or intervention* or strateg*)) or (disseminat* adj2 (implement* or intervention* or strateg*))).mp.
17.	7 or 8 or 9 or 10 or 11 or 12 or 13 or 14 or 15 or 16
18.	6 and 17
19.	limit 18 to yr="2000 -Current"
20.	exp animals/ not humans.sh.
21.	19 not 20
22.	limit 21 to (comment or editorial or letter or news or newspaper article)
23.	21 not 22

Table S2. Evidence of utilisation of implementation concept clusters[23]

	Aveling 2012	Beckett 2012	Klaiman 2014	Russell 2014	McAlearney 2016	Smittenaar 2019	Tucker 2019	Van der Hout 2020	Largey 2021	**Total**
Use evaluative and iterative strategies	+	+	-	+	+	+	+	+	+	8
Provide interactive assistance	-	-	-	+	-	-	+	+	+	4
Adapt and tailor to context	-	+	-	-	+	-	+	+	+	5
Develop stakeholder interrelationships	-	+	+	+	+	+	-	-	+	6
Train and educate stakeholders	-	+	-	+	-	+	+	+	+	6
Support clinicians	-	+	-	+	-	+	-	+	-	4
Engage Consumers	-	+	-	-	-	-	+	+	+	4
Utilize financial strategies	-	-	-	-	-	-	+	-	-	1
Change infrastructure	-	-	-	-	-	-	-	-	-	0
